# Real-Time Moving Object Detection in High-Resolution Video Sensing

**DOI:** 10.3390/s20123591

**Published:** 2020-06-25

**Authors:** Haidi Zhu, Haoran Wei, Baoqing Li, Xiaobing Yuan, Nasser Kehtarnavaz

**Affiliations:** 1 Science and Technology on Micro-system Laboratory, Shanghai Institute of Microsystem and Information Technology, Chinese Academy of Sciences, Shanghai 201800, China; hdzhu@mail.sim.ac.cn (H.Z.); sinowsn@mail.sim.ac.cn (X.Y.); 2 University of Chinese Academy of Sciences, Beijing 100049, China; 3 Department of Electrical and Computer Engineering, University of Texas at Dallas, Richardson, TX 75080, USA; Haoran.Wei@utdallas.edu (H.W.); kehtar@utdallas.edu (N.K.)

**Keywords:** real-time moving object detection, high-resolution object detection, deep neural network moving object detection

## Abstract

This paper addresses real-time moving object detection with high accuracy in high-resolution video frames. A previously developed framework for moving object detection is modified to enable real-time processing of high-resolution images. First, a computationally efficient method is employed, which detects moving regions on a resized image while maintaining moving regions on the original image with mapping coordinates. Second, a light backbone deep neural network in place of a more complex one is utilized. Third, the focal loss function is employed to alleviate the imbalance between positive and negative samples. The results of the extensive experimentations conducted indicate that the modified framework developed in this paper achieves a processing rate of 21 frames per second with 86.15% accuracy on the dataset SimitMovingDataset, which contains high-resolution images of the size 1920 × 1080.

## 1. Introduction

In high-resolution monitoring systems, the capability for real-time processing is needed for object detection applications. The large amount of data required by high-resolution images poses a challenge to achieving real-time moving object detection. In addition, difficulties exist when dealing with complex backgrounds, illumination changes, local motion such as waving trees, dust trailing, camouflage objects, etc.

Existing methods for moving object detection include background subtraction [1,2,3,4,5], frame differencing [6,7], optical flow [8,9,10], ViBe [11,12] and deep learning [13,14,15,16,17,18]. Accuracies are adversely affected when using these methods to achieve real-time detection due to high image resolution and environmental complexities. For example, frame differencing is affected by local motion. When using optical flow methods, its two basic assumptions (constant brightness and slow-motion) are often not met in practice. Furthermore, when using these methods, the category and precise coordinates of each moving object cannot be obtained without the assistance of other algorithms. To some extent, these difficulties can be addressed by combining feature extraction and classification [19,20], but the classification results do not address the situation of moving regions as a group.

Recently, a considerable amount of effort has gone into studying moving object detection with deep learning methods. However, the input of deep neural networks is generally much smaller than a high-resolution image of the size 1920 × 1080. Thus, it is normally difficult to achieve both good speed and accuracy at the same time.

In this paper, the improvement of a previously proposed coarse-to-fine grained framework for moving object detection is discussed in order to achieve real-time detection with high accuracy. Initially, the method to obtain moving regions in the coarse-grained detection stage of the previous framework is modified to increase the computation speed. Then, the backbone of YOLOV3 [21] in the fine-grained detection stage of the previous framework is modified to further increase the computation speed while maintaining good accuracy. Furthermore, a focal loss function [22] is employed to alleviate the imbalance between positive and negative samples.

## 2. Related Works

For moving object detection, different approaches based on the difference in color distribution or pixel intensity have been proposed by researchers, e.g., [1,2,3,4,6,7,8,9,10,23], to eliminate the background in video frames. A widely used algorithm with low computational complexity is frame differencing [6,7], which utilizes the gray level difference between two or three adjacent video frames. However, frame differencing is vulnerable to various interferences caused by local motions and complex scenes. Optical flow methods are based on the assumption that the pixel intensity of objects in the image does not change between successive frames, and also that object motions are relatively slow.

After moving object detection, connected region labeling algorithms can be employed to obtain the coordinates of moving regions. Many works are reported in the literature, e.g., [24,25,26,27,28,29,30], for labeling connected components. However, these methods suffer from high computational complexity. Moreover, these methods have difficulty in merging noise-broken objects. To address this issue, in [31], we presented an efficient algorithm to detect connected regions and merge broken objects at the same time with low computational complexity.

With the recent advancement made in deep learning algorithms (in particular, convolutional neural networks), more effective solutions in terms of higher-accuracy object detection are reported in the literature. More specifically, two-stage detectors [32,33,34,35,36,37] are found to produce accurate detection outcomes. One-stage detectors [21,38,39,40,41,42] are introduced to gain computational efficiency. However, for moving object detection that involves high-resolution images, convolutional neural networks face several limitations [31], including (i) the inability to recognize motion and (ii) the generally much smaller input relative to high-resolution images of the size 1920 × 1080. We proposed the coarse-to-fine grained framework in [31] to address these issues. In that paper, moving regions were obtained during the coarse-grained stage. After that, in order to improve accuracy, a fine-grained detection stage was employed based on the moving regions obtained during the coarse-grained stage. As a result, more accurate coordinates and categories were obtained. However, the issue of achieving a satisfactory trade-off between detection accuracy and computational efficiency still remained.

Thus, in this paper, the objective is to achieve real-time detection together with high accuracy. First, the framework in [31] is used as the starting point. In the coarse-grained detection stage, a more efficient method is considered to obtain moving regions. In order to achieve a higher computation speed, the network structure is modified to a light one. As a result, more accurate coordinates and categories of moving objects are obtained at a higher computation speed in the fine-grained detection stage. Finally, a so-called focal loss function is employed to improve the final outcome. Extensive experimentations are conducted to examine the performance of these modifications for real-time moving object detection on high-resolution (1920 × 1080) images.

More specifically, the contributions of this paper are summarized below:

1. In the coarse-grained detection stage, a more computationally efficient method is developed to obtain moving regions in high-resolution (1920 × 1080) images.
2. The deep neural network is changed to a light one in the fine-grained detection stage to improve computational efficiency.
3. The focal loss function is utilized to improve accuracy while alleviating the imbalance between positive and negative samples.

The rest of the paper is organized as follows: Section 3 discusses the details associated with the above steps. In Section 4, the experimental results are stated and discussed. The paper is then concluded in Section 5.

## 3. Improved Moving Object Detection Framework

**Figure 1:**
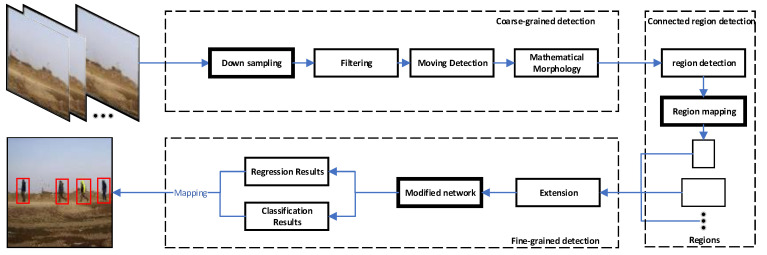
Developed a modified moving object detection framework.

In this section, the improvements made to our previously developed framework for moving object detection are discussed. Figure 1 illustrates the modules of the improved framework developed in this paper. The modifications are highlighted with bold boxes. Let us begin with the coarse-grained detection block or stage, which consists of moving object detection with downsampling together with low-pass filtering and morphology filtering (opening operation). Then, adjustments are made to the framework in [31] to obtain connected region detection by mapping the coordinates of moving regions to the original image. In addition, in order to obtain more complete moving regions, the regions are extended. After cropping, the regions obtained in the coarse-grained detection stage are fed into a modified network to enable fine-grained detection. As moving objects are initially detected in the coarse-grained detection stage, in the fine-grained detection stage, objects occupy larger areas in the regions. Hence, the input size of the deep neural network becomes 320 × 320, or smaller than the size 416 × 416 used in YOLOV3 [21].

Finally, according to the positional relationship between objects and moving regions, the coordinates on the original image (1920 × 1080) are obtained. Basically, the framework mainly contains a moving detection module with downsampling, a connected region extraction module with region mapping and an object detection module with a light backbone.

### 3.1. Coarse-Grained Detection Stage

In the coarse-grained detection stage, low-pass filtering and morphology filtering are performed to reduce the ill effects of noises. First, video frames are resized by downsampling to reduce the amount of data. Then, each resized image is filtered by low-pass filtering to eliminate high-frequency noises. After that, a moving detection algorithm is applied to two consecutive frames for detecting motion. Finally, a morphology filtering operation (opening operation) is performed to further suppress the ill effects of noises. Furthermore, the outcome is refined by fine-grained detection.

Due to the considerable amount of data associated with high-resolution scenes, frame differencing is used here for moving object detection, as it is simple to implement and responsive to nearly all movements. In [31], frame differencing was also employed because of its low computational complexity and high sensitivity to movements, and the comparison with other algorithms such as GMM showed its effectiveness. In the coarse-grained detection stage, in order to reduce the ill effects of noises, low-pass filtering and morphology filtering are performed. Note that frame differencing, low-pass filtering and morphology filtering are conducted pixel by pixel. Therefore, the process is still time-consuming and requires a considerable amount of runtime. Hence, in this paper, downsampling is considered to achieve higher computational efficiency, reducing the amount of computation. For downsampling, the Nearest Neighbor Interpolation algorithm is employed, which is computationally efficient and easy to implement. Furthermore, the moving regions are refined in the fine-grained detection stage.

With the connected region detection algorithm, the coordinates of each moving region on the downsampled image are obtained. However, after downsampling, small objects in the image would appear smaller. To address the adverse impact of downsampling for small objects, moving regions in the downsampled image obtained by the coarse-grained detection stage are mapped to the original size image for the fine-grained detection stage. In other words, the coordinates of moving regions on the original size image are found before applying the fine-grained detection stage.

Assuming that the upper-left and lower-right coordinates of the moving region on the downsampled image are (*x*_0_, *y*_0_) and (*x*_1_, *y*_1_), respectively, let the coordinates of the corresponding regions on the original image be represented by [(*x_min_*, *y_min_*), (*x_max_*, *y_ma_*_x_)], as defined in Equation (1), where scale denotes the scale of downsampling, which is considered to be 5 in this work. Furthermore, the regions on the original image are expanded to make sure a complete moving region for the fine-grained detection stage can be obtained.

(1) $$x_{min}=x_{0}*scale,y_{min}=y_{0}*scale\;x_{max}=x_{1}*scale,y_{max}=y_{0}*scale$$

### 3.2. Fine-Grained Detection Stage

#### 3.2.1. Light Deep Neural Network Backbone

In [31], MTiny YOLOV3 with a small input size and fewer anchors was employed in the fine-grained detection stage to gain a faster computation speed. Although a faster speed was obtained, the accuracy dropped considerably (from 88.59% to 80.77%). Therefore, Mobilenet, the deep neural network proposed in [43], is used by substituting the backbone of YOLOV3 (Mobilenet-YOLOV3) in the fine-grained detection stage. Mobilenet-YOLOV3 makes predictions based on three scales with a light backbone, with Mobilenet extracting the features. In Mobilenet, depthwise separable convolution consists of depthwise convolution and pointwise convolution to gain computational efficiency. Since objects occupy a large area of the moving regions obtained in the coarse-grained detection stage, the use of a complex network is eased and a light backbone can still achieve favorable accuracy.

#### 3.2.2. Modified Loss Function

For the one-stage detector, one critical issue has an ill effect on the detection accuracy due to the imbalance in the proportion of positive and negative samples. In order to alleviate this imbalance and make the network focus on the samples simultaneously, the focal loss function [22] defined in Equation (2) is employed for training, where *y’* denotes the predicted value and *y* is the label. In Equation (2) *a* and *b* are utilized to balance the importance of positive and negative samples and make the network pay more attention to the samples separately. For the results reported in this paper, *a* is set to 0.75 and *b* is set to 2. Therefore, with focal loss, higher detection accuracy is achievable with a similar inference computation speed.

(2) $$L_{focal}=\{\begin{array}{c} -a{\left(1-y^{\prime }\right)}^{b}logy^{\prime },\;y=1\\ -\left(1-a\right){y^{\prime }}^{b}log\left(1-y^{\prime }\right),y=0 \end{array}$$

## 4. Experimental Results and Discussion

In this section, the results of extensive experimentations conducted show the effectiveness of the modified framework. This framework was implemented on a standard GPU (NVIDIA GeForce GTX 1080TI). The dataset used is SimitMovingDataset, the same as the one used in [31] (1920 × 1080). This dataset incorporates various challenging scenarios, such as local motion, camouflage, multi-scale objects, occlusion, illumination changes, complex background and dust trailing. The mean Average Precision (mAP) discussed in [31] is utilized here to measure the detection accuracy in order to incorporate both the regression and classification aspects of the developed framework. In addition, a commonly used metric AP_75_ is computed. If not otherwise specified in the paper, the detection accuracy is measured based on mAP.

### 4.1. Downsampling in Coarse-Grained Detection Stage

In order to examine the effectiveness of the framework with downsampling, the original framework in [31] was also tested to compare the performance in terms of runtime and accuracy, as evidenced in Table 1. In Table 1, blur size is the parameter of the low-pass filtering, which determines the degree of filtering. Larger sizes lead to more blurring in the output image and more computational complexity. Moreover, the threshold is the parameter associated with frame differencing, which affects movement detection. Small thresholds lead to pixels with a smaller difference in intensity values between two consecutive frames to be regarded as moving pixels. For the results reported in the following parts, blur size is set to 11 and the threshold is set to 15. Compared with the results of the framework in [31] (see Table 2), the modified framework with downsampling provided better execution time with higher accuracy.

**Figure 2:**
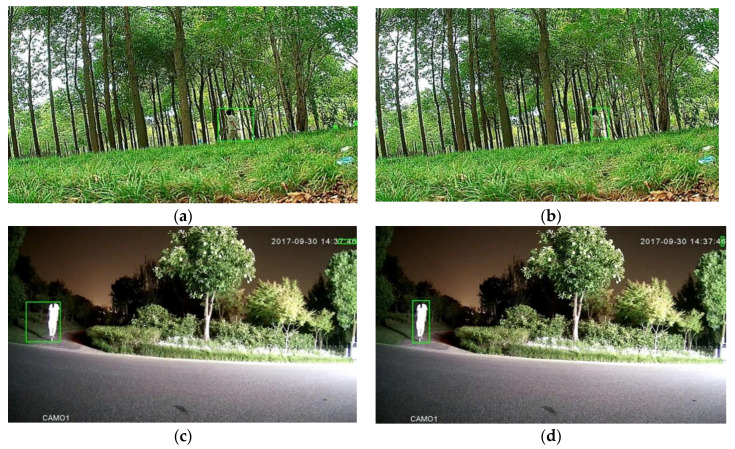

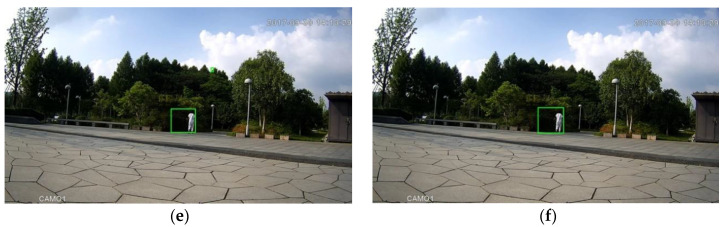
Outcomes of the coarse-grained detection without downsampling (**a,c,e**) and with downsampling (**b,d,f**). The left column and right column correspond to the same images.

**Figure 3:**
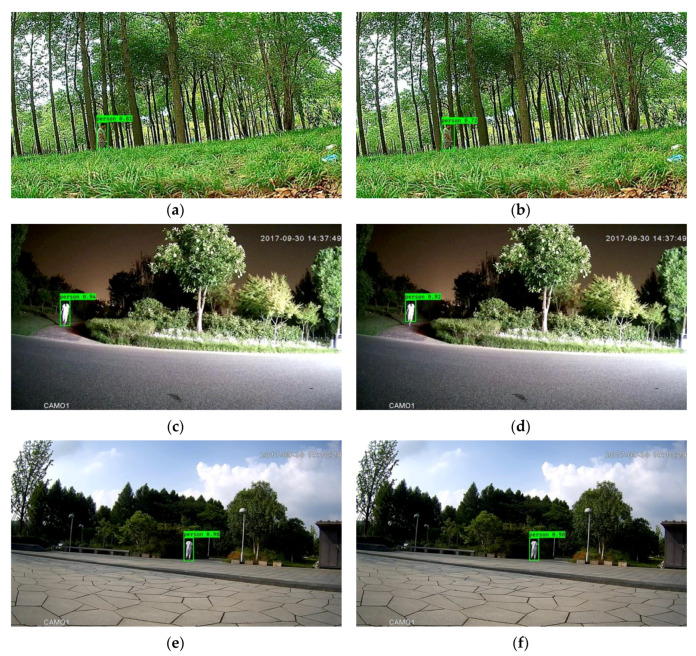
Outcomes of the framework without downsampling (**a,c,e**) and with downsampling (**b,d,f**). The left column and right column correspond to the same images.

As an example of the effectiveness of downsampling, Figure 2 illustrates the outcome of the coarse-grained detection stage consisting of moving object detection with low-pass filtering and morphology filtering (the left column), as well as the outcome with downsampling (the right column). From this figure, it can be observed that the coarse-grained detection with downsampling is comparable. This observation is further supported by the outcome shown in Figure 3, which is the output of the entire framework with downsampling, Mobilenet backbone and focal loss mentioned in Section 4.2.

### 4.2. Improvements in Fined-Grained Detection Stage

#### 4.2.1. Light Backbone

From the results shown in Section 4.1, it can be observed that a higher speed was obtained with downsampling, but the accuracy dropped considerably when the speed was much higher. Therefore, it was not possible to achieve real-time detection with high accuracy using only downsampling. In order to further improve the computational efficiency, a light backbone, Mobilenet, was employed to extract the features.

To explore the computational efficiency of the framework with a light backbone, experiments were conducted to compare the original framework [31] and the modified framework with a light backbone. The results reported in Table 3 show that the modified framework performed approximately 1.67 times faster than the framework with YOLOV3 [31]. Furthermore, the accuracy dropped by only 0.84%. Compared with the framework with MTiny YOLOV3 [31], the results of the framework with Mobilenet-YOLOV3 show advantages in terms of accuracy with fast runtime. From Table 3, it can also be seen that it is difficult for MTiny YOLOV3 (input image size of 96*96) to obtain high accuracy regression bounding boxes.

#### 4.2.2. Further Improvement for Downsampling

**Figure 4:**
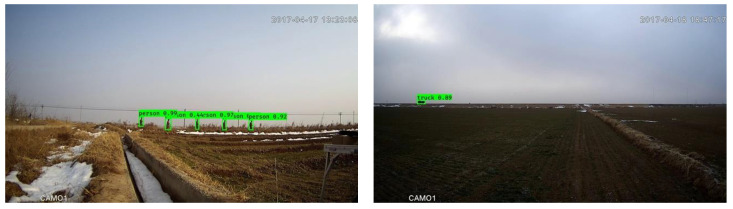

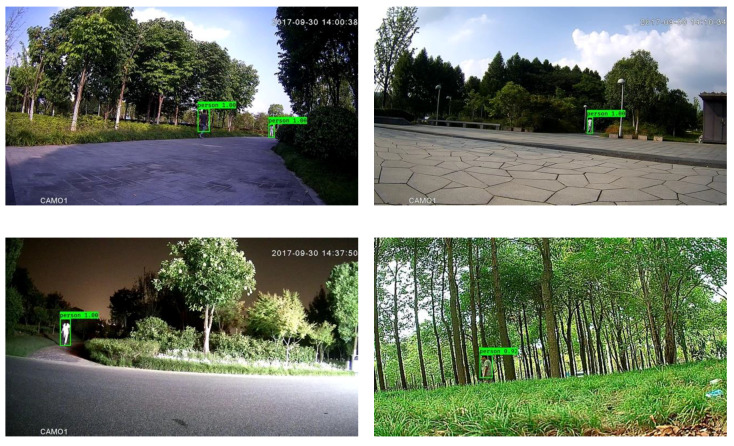
Outcomes of the framework with Mobilenet-YOLOV3 and downsampling.

In order to obtain higher computational efficiency, downsampling was employed in the framework with Mobilenet-YOLOV3. As shown in Table 4, compared with the modified framework without downsampling, it can be observed that the modified framework with downsampling obtained approximately 1.40 times speedup. In addition, as shown in Table 3 and Table 4, the modified framework outperformed the original framework with MTiny YOLOV3 in terms of accuracy, 4.12% higher with similar execution time. The importance of the framework with a light backbone and downsampling is in conducting real-time detection with favorable accuracy. Furthermore, from a qualitative perspective, Figure 4 illustrates the effectiveness of the modified framework with Mobilenet-YOLOV3 and downsampling.

#### 4.2.3. Focal Loss

**Figure 5:**
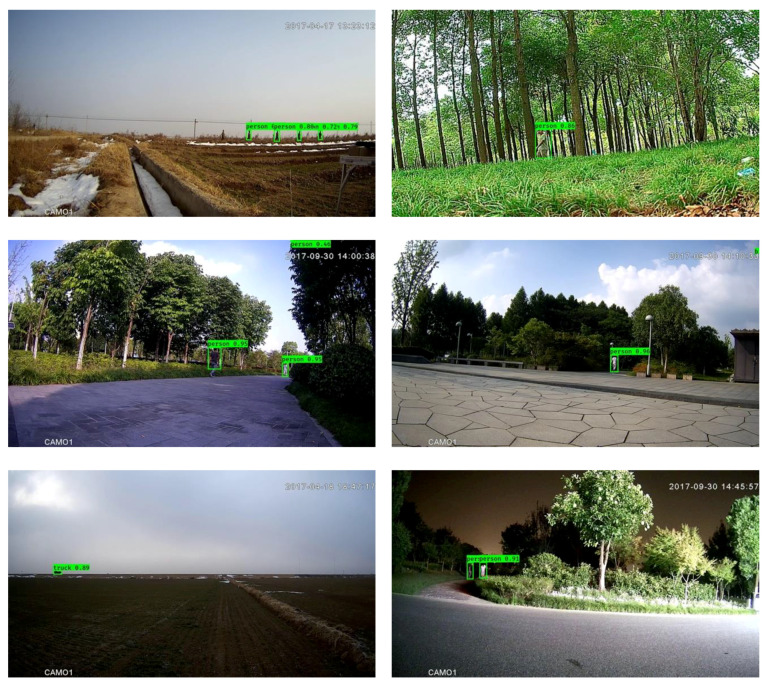
Outcomes of the framework with focal loss and downsampling.

Table 3 and Table 4 provide the advantages of the modified framework with Mobilenet-YOLOV3 over that with YOLOV3 or MTiny YOLOV3 in terms of establishing a balance between computational efficiency and detection accuracy. To address the imbalance of negative and positive samples, the focal loss function was employed. The number of anchors per grid was altered to 2 to achieve a better balance between positive and negative samples. To study the effectiveness of focal loss, ablation experiments were conducted on the framework with Mobilenet-YOLOV3. Different components were omitted in the framework to observe the effectiveness of each component, including focal loss and downsampling. As shown in Table 4 and Table 5, one sees the obvious benefits of focal loss, as confirmed visually in Figure 5. In addition, from Table 5, the detection accuracy (AP_75_) is seen to be similar for the modified framework with and without downsampling. In other words, it is seen that downsampling with an extension operation (in the fine-grained detection stage) would not have an adverse impact on the samples that are easier detected (corresponding to more accurate predicted bounding boxes) based on Mobilenet-YOLOV3 and focal loss.

### 4.3. Discussion on Input Size

**Figure 6:**
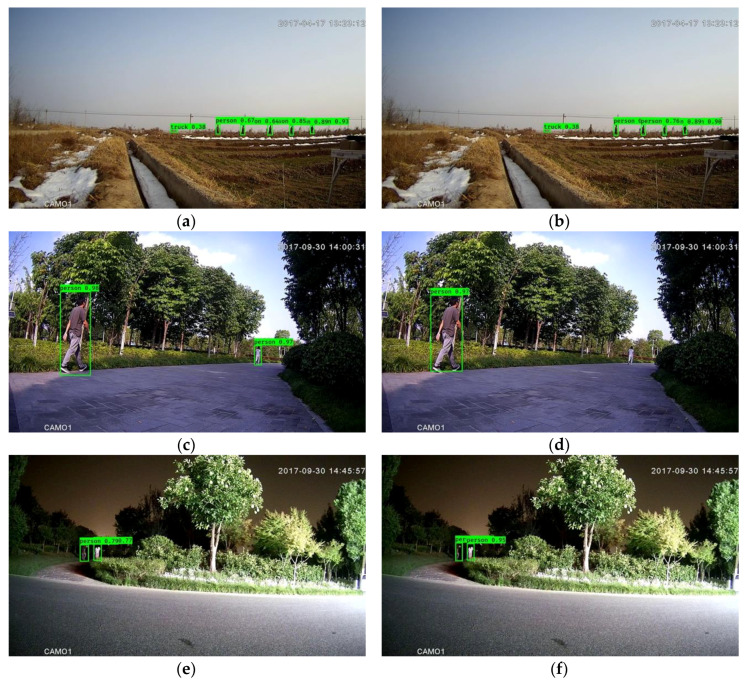
Outcomes of the framework with 416 × 416 input size without downsampling (**a,c,e**) and with downsampling (**b,d,f**). The left column and right column correspond to the same images.

**Figure 7:**
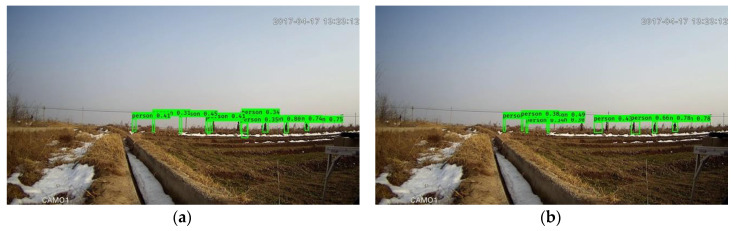

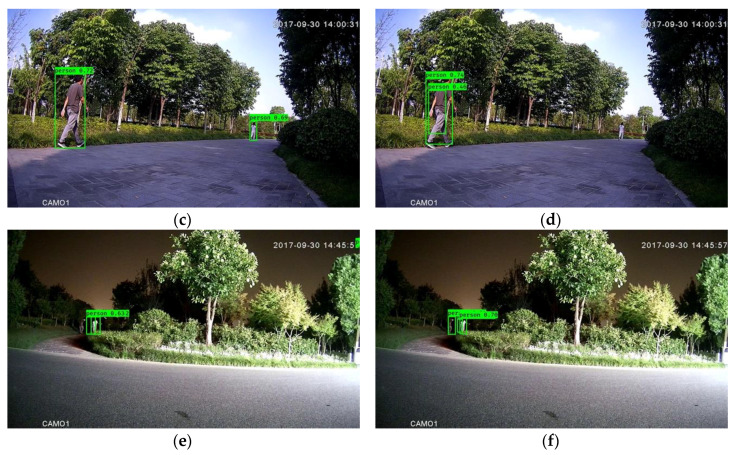
Outcomes of the framework with 96*96 input size without downsampling (**a,c,e**) and with downsampling (**b,d,f**). The left column and right column correspond to the same images.

**Figure 8:**
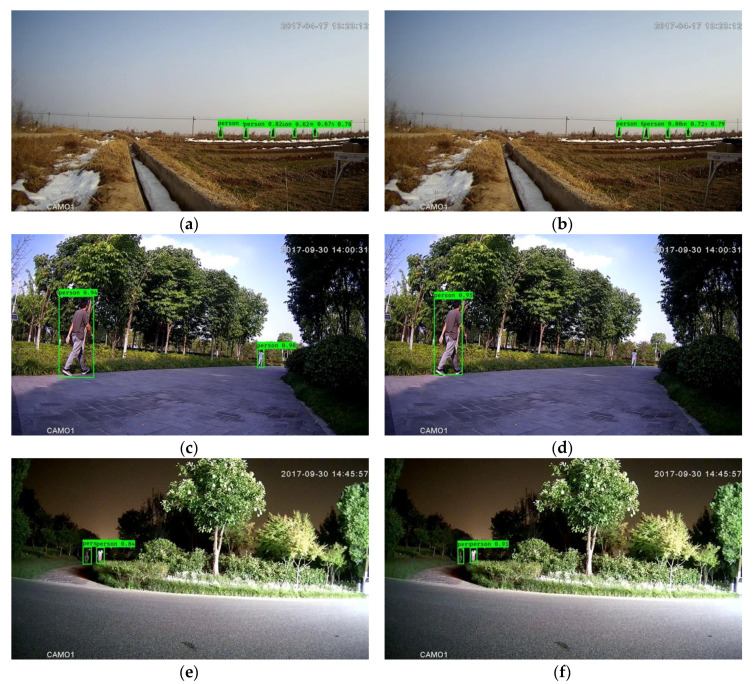
Outcomes of the framework with 320 × 320 input size without downsampling (**a,c,e**) and with downsampling (**b,d,f**). The left column and right column correspond to the same images.

Generally, the input size of the network has a certain influence on the detection accuracy and execution time. In the experimentations provided in this subsection, several input sizes were examined. For fine-grained detection, the detected regions are obtained after the coarse-grained detection. Objects occupy a large area, and thus, a large input size is not necessary. In addition, with the size of 320 × 320, the imbalance of positive and negative samples is alleviated to some degree. As shown in Table 6, an input size of 320 × 320 shows advantages in terms of detection accuracy and computational efficiency, which is further verified visually in Figure 6, Figure 7 and Figure 8, with the outcome in Figure 8 being better than those in Figure 6 and Figure 7. Furthermore, as seen in Table 6, the detection accuracy (AP_75_) using a 96 × 96 input size with downsampling is not inferior to that without downsampling. In other words, downsampling with an extension operation (in the fine-grained detection stage) would not have an adverse impact on the samples that are more easily detected for obtaining accurate bounding boxes.

### 4.4. Ablation Analysis

**Figure 9:**
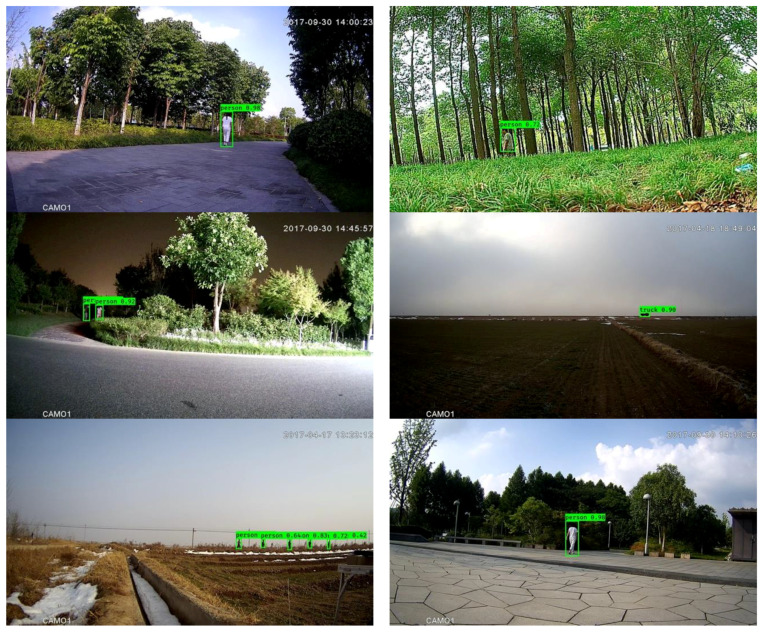
Outcomes of the entire framework.

In the experimentations reported in this subsection, each modified component, including downsampling, light backbone and focal loss, was omitted separately to examine its effectiveness. The results obtained are shown in Table 7. As seen in Figure 9, the final outcome of the framework with Mobilenet-YOLOV3, focal loss and downsampling worked well on the high-resolution video frames (1920 × 1080).

## 5. Conclusions

This study addresses the problem of real-time moving object detection with high accuracy on high-resolution scenes in the presence of different kinds of noises. Modifications are made to the framework previously reported in [31] in order to obtain a better balance between accuracy and speed. The modified framework includes Mobilenet-YOLOV3, downsampling and the use of the focal loss function. Its effectiveness is verified by extensive experimentations. In order to improve the performance in terms of detection accuracy and computational efficiency, the backbone of YOLOV3 is altered to Mobilenet, achieving 1.67 times faster computation speed with similar accuracy. Combined with downsampling, it achieves 2.33 times faster computation speed than the original framework with YOLOV3. Finally, the use of focal loss and a suitable number of anchors per grid leads to a higher speedup. In summary, the modified framework developed in this paper is able to achieve approximately 21 FPS (Frames Per Second) processing rate with 86.15% accuracy, which is 2.33 times faster than the framework using YOLOV3.

## Figures and Tables

**Table 1:** Results of the original framework with downsampling.

Blur size	Threshold	Time (s)	mAP (%)	AP_75_ (%)
11	15	0.088	85.84	72.04
11	20	0.078	81.32	69.01
11	25	0.070	74.61	64.34
15	15	0.071	77.59	66.94
9	20	0.088	84.50	69.78

**Table 2:** Results of different thresholds T0 [31] representing the maximum number of regions detected sequentially on one video frame.

T0	mAP (%)	Time (s)
50	88.59	0.112
10	84.24	0.105
8	81.71	0.103
6	73.34	0.093
4	66.13	0.081
0	48.24	0.059

**Table 3:** Results of the frameworks with different networks in the fine-grained detection stage.

Framework	Time (s)	mAP (%)	AP_75_ (%)
Original framework with YOLOV3	0.112	88.59	73.69
Original framework with MTiny YOLOV3	0.043	80.77	36.44
Framework with Mobilenet-YOLOV3	0.067	87.85	70.26

**Table 4:** Results of the framework based on Mobilenet-YOLOV3 with and without downsampling.

Network	Downsampling	Time (s)	mAP (%)	AP_75_ (%)
Mobilenet-YOLOV3	N	0.067	87.85	70.26
Mobilenet-YOLOV3	Y	0.048	84.10	64.04

**Table 5:** Results of the modified framework with focal loss with and without downsampling.

Framework	Downsampling	Time (s)	mAP (%)	AP_75_ (%)
Modified framework with Focal loss	N	0.067	88.59	71.42
Modified framework with Focal loss	Y	0.048	86.15	71.61

**Table 6:** Results of the framework with focal loss, with and without downsampling on different input sizes.

Input Size	Downsampling (Y/N)	Time (s)	mAP (%)	AP_75_ (%)
416*416	N	0.079	86.62	67.97
416*416	Y	0.048	82.03	66.65
320*320	N	0.067	88.59	71.42
320*320	Y	0.048	86.15	71.61
96*96	N	0.060	68.78	21.62
96*96	Y	0.031	63.77	29.44

**Table 7:** Results of the framework with different components.

Network	Downsampling	Focal Loss	Time (s)	mAP (%)	AP_75_ (%)
YOLOV3	N	N	0.112	88.59	73.69
MTiny YOLOV3	N	N	0.043	80.77	36.44
Mobilenet-YOLOV3	N	N	0.067	87.85	70.26
Mobilenet-YOLOV3	Y	N	0.048	84.10	64.04
Mobilenet-YOLOV3	N	Y	0.067	88.59	71.42
Mobilenet-YOLOV3	Y	Y	0.048	86.15	71.61
